# Autophagy-Related Protein ATG18 Regulates Apicoplast Biogenesis in Apicomplexan Parasites

**DOI:** 10.1128/mBio.01468-17

**Published:** 2017-10-31

**Authors:** Priyanka Bansal, Anuj Tripathi, Vandana Thakur, Asif Mohmmed, Pushkar Sharma

**Affiliations:** a Eukaryotic Gene Expression Laboratory, National Institute of Immunology, New Delhi, India; b International Centre for Genetic Engineering and Biotechnology, New Delhi, India; Albert Einstein College of Medicine

**Keywords:** *Plasmodium falciparum*, *Toxoplasma gondii*, apicoplast, autophagy, cell trafficking, phosphoinositides

## Abstract

Mechanisms by which 3′-phosphorylated phosphoinositides (3′-PIPs) regulate the development of apicomplexan parasites *Plasmodium falciparum* and *Toxoplasma gondii* are poorly understood. The catabolic process of autophagy, which is dependent on autophagy-related proteins (ATGs), is one of the major targets of 3′-PIPs in yeast and mammals. In the present study, we identified autophagy-related protein ATG18 as an effector of 3′-PIPs in these parasites. *P*. *falciparum* ATG18 (PfATG18) and *T*. *gondii* ATG18 (TgATG18) interact with 3′-PIPs but exhibited differences in their specificity of interaction with the ligand PIP. The conditional knockdown of *T*. *gondii* or *P*. *falciparum* ATG18 (Tg/PfATG18) impaired replication of parasites and resulted in their delayed death. Intriguingly, ATG18 depletion resulted in the loss of the apicomplexan parasite-specific nonphotosynthetic plastid-like organelle apicoplast, which harbors the machinery for biosynthesis of key metabolites, and the interaction of ATG18 to phosphatidylinositol 3-phosphate (PI3P) was critical for apicoplast inheritance. Furthermore, ATG18 regulates membrane association and apicoplast localization of ATG8. These findings provide insights into a novel noncanonical role of ATG18 in apicoplast inheritance. This function of ATG18 in organelle biogenesis is unprecedented in any organism and may be conserved across most apicomplexan parasites.

## IMPORTANCE

Typically, autophagy is a catabolic process utilized by cells for their survival upon encountering nutrient-limiting conditions. The autophagy machinery is very tightly regulated, and autophagy-related genes (ATGs) play a pivotal role in this process. In the present study, we report a novel noncanonical function of autophagy-related protein ATG18 in inheritance of the nonphotosynthetic plastid-like organelle apicoplast in apicomplexan parasites *Plasmodium* and *Toxoplasma*. ATG18 depletion in these parasites resulted in “delayed death,” which was the result of loss of apicoplast and impaired parasite division. Pf/TgATG18 interact with 3′-phosphorylated PIPs, which guide their cellular localization in the parasite, which is essential for their function.

## INTRODUCTION

*Plasmodium falciparum* and *Toxoplasma gondii* are obligate intracellular protozoan parasites that adapt to their specific host environments for their proliferation. These parasites belong to the phylum *Apicomplexa*, which diversified from alveolate ancestors. While there are similarities in some processes such as host cell invasion, these parasites cause infection by undergoing differentiation in different host cells. During intraerythrocytic development, *Plasmodium* matures from rings to trophozoites, followed by asexual division, yielding up to ~30 merozoites per schizont. The lytic cycle of *T. gondii* tachyzoites causes disease symptoms as they are the rapidly dividing form of the parasite (1). Most apicomplexan parasites possess a plastid-like organelle called the apicoplast, which was acquired by secondary endosymbiosis and is surrounded by four membranes (2) and is segregated during cell division (3). The apicoplast is essential for parasite survival and contains key pathways involved in biosynthesis of important metabolites (4).

Phosphoinositides (PIPs) that are generated by the action of phosphoinositide kinases (PIKs) on phosphatidylinositol (5) regulate diverse critical processes in these parasites (6–10). However, mechanisms by which PIPs regulate parasitic processes are poorly understood. Phosphatidylinositol 3-kinases (PI3Ks) play a critical role in autophagy of most organisms via ATG18 or its homologues like WIPI1 (WD repeat domain, phosphoinositide interacting 1), which interact with 3′-PIPs (11). *In silico* analysis suggested that apicomplexan parasites have simplified autophagy pathways, as several autophagy-related genes are absent from them (12–14). Most studies have centered around ATG8 or its regulators in these parasites (13–17), but the function of most autophagy-related genes remains poorly understood. Here, we have identified a homologue of ATG18 in *P. falciparum* and *Toxoplasma gondii* as an effector of 3′-phosphorylated PIPs. Incidentally, a recent study reported artemisinin-resistant mutations in *P. falciparum* ATG18 (PfATG18) (18), although the contribution of these mutations, if any, to drug resistance has not be determined. In the present study, we demonstrate that *P. falciparum* and *T*. *gondii* ATG18 (Pf/TgATG18) are involved in apicoplast biogenesis and depletion of ATG18 in *P. falciparum* and *T. gondii* resulted in “delayed death” phenotype, a phenomenon previously associated with the loss of the plastid (19).

## RESULTS

### Pf/TgATG18 are important for parasite growth and replication.

BLAST searches using either yeast or human ATG18/WIPI1 sequences against PlasmoDB resulted in identification of a gene identified as PF3D7_1012900, which is consistent with previous studies (13). Since we also wanted to identify the homologue in closely related apicomplexan *Toxoplasma*, the PfATG18 amino acid sequence was used to search the ToxoDB database. TGGT1_220160 exhibited significant homology over almost the entire length of the protein (41%) as indicated by the ClustalW-based alignment, and structure-based comparison indicated that all seven blades of the ATG18 β-propeller may be conserved in *Plasmodium falciparum* or *Toxoplasma gondii* ATG18 (Pf/TgATG18) (see Fig. S1 in the supplemental material). *Toxoplasma* seems to possess another ATG18-related gene, TGGT1_288600; however, it exhibited lower sequence homology (27%) with PfATG18 (not shown here). Therefore, further studies were performed with TGGT1_220160, which was named *T*. *gondii* ATG18 (TgATG18).

10.1128/mBio.01468-17.1 FIG S1  Sequence comparison of PfATG18 and TgATG18 with ScATG18. Download FIG S1, PDF file, 0.2 MB. Copyright © 2017 Bansal et al. 2017 Bansal et al. This content is distributed under the terms of the Creative Commons Attribution 4.0 International license.

**FIG 1:**
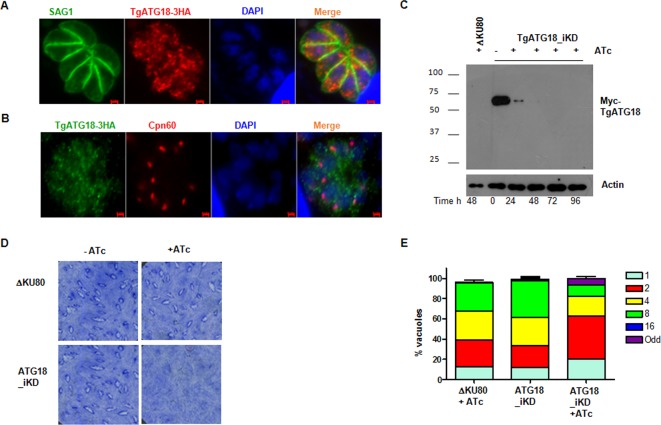
Depletion of TgATG18 causes delayed death of *T. gondii*. (A and B) Immunofluorescence assays (IFAs) were performed on TgATG18-3HA parasites using antibodies against HA tag and SAG1 (A) and with Cpn60 (B). (C) Depletion of TgATG18 in *Toxoplasma gondii*. TgATG18-iKD parasites (see Fig. S2A and B in the supplemental material) or ΔKu80 parasites were treated (+) with anhydrotetracycline (ATc) for the duration (in hours) indicated below the bottom gel. Western blotting of parasite lysate revealed that inducible Myc-labeled TgATG18 (Myc-TgATG18) migrates to the expected position, and the addition of ATc resulted in its depletion. The positions of molecular mass markers (in kDa) are shown to the left of the gel. (D) Plaque assays were carried out by infecting HFF monolayer with ΔKu80 or TgATG18-iKD parasites in the presence (+) or absence (−) of ATc for 7 days. (E) Depletion of TgATG18 impaired parasite intracellular replication. TgATG18-iKD or ΔKu80 parasites were preincubated for 48 h with ATc and subsequently allowed to invade fresh HFFs in the presence or absence of ATc. The number of parasites per vacuole was determined after 24 h. Data represent means ± standard errors of the means from three experiments, and at least 200 vacuoles were counted for each condition.

TgATG18 was tagged at the C terminus with hemagglutinin (HA) to determine its localization. Immunofluorescence assays (IFAs) revealed that TgATG18 is present in punctate vesicular structures that were spread throughout the parasite cytoplasm (Fig. 1A). Unlike TgATG8, which has been localized to the apicoplast (16), we did not find significant colocalization of TgATG18 with apicoplast protein Cpn60 (Fig. 1B).

In order to investigate the function of TgATG18, an inducible knockdown was generated in *Toxoplasma* (TgATG18-iKD) using a tetracycline-based transactivator system (20) (Fig. S2A and B). Western blotting indicated that TgATG18 levels were significantly depleted after 24 h of anhydrotetracycline (ATc) treatment (Fig. 1C), which was also confirmed by IFA (Fig. S2C). Plaque assays, which are used to assess the lytic cycle of *Toxoplasma*, revealed that no visible plaques were formed by TgATG18-iKD parasites after 7 days of 1 μM ATc treatment (Fig. 1D). However, intracellular growth assays revealed only a marginal difference in parasite growth during the first lytic cycle (24 h after ATc treatment) (Fig. S2D). In sharp contrast, in the next cycle (72 h after ATc treatment), almost 60% of the vacuoles in ATc-treated TgATG18-iKD contained two to four parasites, while untreated parasites or parental strain continued to replicate as well as wild-type parasites did (Fig. 1E). The observation that parasite growth was mainly altered after the first lytic cycle was indicative of a delayed death phenotype.

10.1128/mBio.01468-17.2 FIG S2  Generation and analysis of TgATG18-iKD parasites. Download FIG S2, PDF file, 0.2 MB. Copyright © 2017 Bansal et al. 2017 Bansal et al. This content is distributed under the terms of the Creative Commons Attribution 4.0 International license.

**FIG 2:**
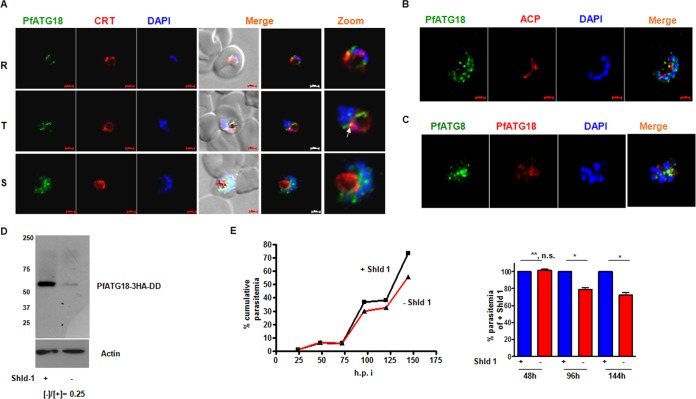
PfATG18 depletion causes delayed death of *P*. *falciparum*. (A) IFAs were performed on rings (R), trophozoites (T), and schizonts (S) of PfATG18-3HA-DD parasites using anti-HA antibody and antisera against a food vacuole membrane protein PfCRT. PfATG18 was localized in vesicles, which increased in number as the parasite matured to trophozoites (T) and schizonts (S), and some of the vesicles were present around the food vacuole (white arrow). (B and C) IFAs were performed on PfATG18-3HA-DD schizonts using anti-HA antibody to detect PfATG18 and antibodies against acyl carrier protein (ACP) (B) and PfATG8 (C). (D) Knockdown of PfATG18 in *P. falciparum*. Western blotting was performed on lysates of PfATG18-3HA-DD parasites after Shld-1 withdrawal using anti-HA antibody. PfATG18-3HA-DD was observed at the expected migration position and was significantly downregulated upon Shld-1 withdrawal. Actin was used as a loading control. (E) PfATG18-3HA-DD parasites were synchronized, and ring-stage parasites were used for growth assays in the presence (+) or absence (−) of Shld-1. Parasite growth was monitored by analysis at the indicated time points (in hours postinfection [h.p.i.]). (Right) The parasitemia at 48, 96, and 144 h from three independent experiments similar to the experiment shown in the graph to the left (means ± standard errors of the means [*n* = 3]). Values that are significantly different by *t* test are indicated by bars and asterisks as follows: *, *P* < 0.001 **, P > 0.05. Values that are not significantly different (n.s.) by *t* test are indicated.

We also investigated the role of PfATG18 in the development of *P. falciparum* and used a conditional knockdown strategy involving FKBP destabilization domain (DD) (21) (Fig. S3A and B). In addition, PfATG18 was fused to a 3×HA tag at the C terminus. Immunofluorescence assays revealed that PfATG18 resides in vesicular structures (Fig. 2A), and PfATG18-positive vesicles increased with *P. falciparum* maturation (Fig. 2A), which corroborated well with increases in PfATG18 expression in mature trophozoites and schizonts (Fig. S3D). It was evident that some PfATG18 vesicles were in close vicinity of the food vacuole which was stained with an antibody against food vacuole membrane protein *P*. falciparum CRT (PfCRT) (Fig. 2A). While there was no apparent colocalization of PfATG18 with the apicoplast (Fig. 2B), some PfATG18-labeled vesicles were detected near the branching apicoplast (Fig. 2B) and in proximity of vesicles containing downstream autophagy protein PfATG8 (Fig. 2C), which was transiently localized to the apicoplast in previous studies (13, 22) (see Fig. 6A).

10.1128/mBio.01468-17.3 FIG S3  Generation of PfATG18-3HA-DD parasites for PfATG18 depletion and localization of PfATG18. Download FIG S3, PDF file, 0.2 MB. Copyright © 2017 Bansal et al. 2017 Bansal et al. This content is distributed under the terms of the Creative Commons Attribution 4.0 International license.

The removal of the DD-stabilizing ligand Shield-1 (Shld-1) resulted in a significant reduction of PfATG18 expression as a result of proteosomal degradation (Fig. 2D). While parasite growth for the first cycle (48 h) was almost unchanged, Shld-1 removal caused increasing impairment in the subsequent cycles (Fig. 2E and Fig. S3C). These observations were typical of the delayed death phenotype observed for *P. falciparum* (23).

### ATG18 is involved in apicoplast inheritance.

**FIG 3:**
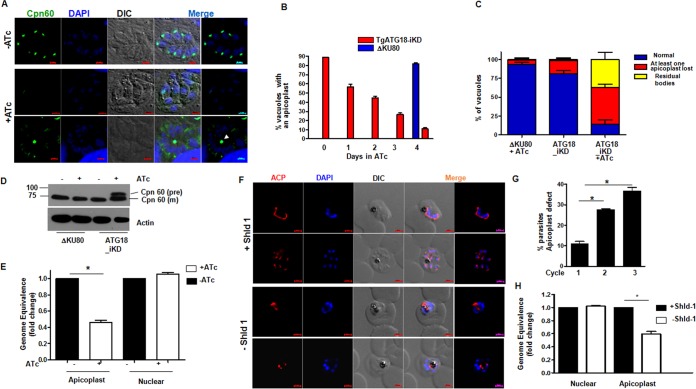
Tg/PfATG18 is involved in apicomplexan apicoplast inheritance. (A) IFA was performed on ΔKu80 or TgATG18-iKD parasites pretreated with ATc for 48 h, followed by additional treatment for 2 days to detect apicoplast using an antibody against apicoplast resident protein Cpn60. Control ΔKu80 parasites or untreated TgATG18-iKD parasites exhibited apicoplast segregation to individual tachyzoites. In contrast, ATc-treated TgATG18-iKD parasites either lacked apicoplast, or it was found in the residual body (white arrowhead). DIC, differential interference contrast. (B) TgATG18-iKD or ΔKu80 parasites were treated with ATc for the indicated time (in days), and IFA was performed using Cpn60 antibody. Vacuoles that possessed at least one parasite with an apicoplast were counted. (C) Quantification of vacuoles containing all apicoplasts (blue) in which at least one apicoplast was lost (red) or an apicoplast was found in the residual body (yellow) in parasites that were treated with ATc for 96 h. Data are means ± standard errors of the means from three independent experiments, and at least 200 vacuoles were counted for each condition. (D) ΔKu80 or TgATG18-iKD parasites were treated with ATc for 96 h as described above for panel A. Western blotting was performed on parasite lysates using anti-Cpn60 antibody. In all cases, mainly the mature (m) Cpn60 was found, but when TgATG18-iKD parasites were treated with ATc, the precursor Cpn60 (pre) was observed. (E) Quantitative PCR was performed using total DNA from TgATG18-iKD parasites treated with ATc for 48 h and primers specific to nuclear and apicoplast genes. Fold change in apicoplast and nuclear genome is provided. Values are means plus standard errors of the means from three experiments. Values that are significantly different (*P* < 0.001) by analysis of variance (ANOVA) are indicated by a bar and asterisk. (F) PfATG18-3HA-DD parasites were synchronized, and ring-stage parasites were cultured in the presence or absence of Shld-1. Early or late schizonts after the first, second, and third cycle (shown here) were used for IFA using an antibody against ACP, an apicoplast resident protein. In the presence of Shld-1, apicoplast branching was visible in early division and subsequently each of the newly formed merozoites received an individual apicoplast. Shld-1 removal resulted in stunted branching of ACP and impaired segregation. (G) Quantification of parasites with abnormal apicoplast branching and segregation as described above after the removal of Shld-1 for the indicated number of cycles. Values that are significantly different (*P* < 0.001) by ANOVA are indicated by a bar and asterisk. (H) Quantitative PCR was performed using total DNA from PfATG18-3HA-DD parasites cultured in the absence or presence of Shld-1 for 144 h and primers specific to nuclear genome and apicoplast genes. Change in expression (fold change in apicoplast and nuclear genome) is shown on the *y* axis. Values are means ± standard errors of the means from three experiments. Values that are significantly different (*P* < 0.001) by ANOVA are indicated by a bar and asterisk.

Since the “delayed death” phenotype has been associated with the loss of apicoplast (19), the role of TgATG18 in apicoplast biogenesis was investigated. In the absence of ATc, parasite staining with anti-Cpn60, a nucleus-encoded apicoplast luminal chaperone, expectedly revealed an apicoplast associated with each tachyzoite. TgATG18 depletion resulted in apicoplast loss in a significant number of parasites, as Cpn60 staining was either diffused, missing, or found in the residual bodies (Fig. 3A and B). Apicoplast was lost in a substantial number of parasites within 24 to 48 h of ATc treatment, and less than 10% of vacuoles had parasites that possessed an apicoplast after 96 h (Fig. 3B and C). Furthermore, Western blotting revealed the accumulation of the precursor form of Cpn60 upon TgATG18 depletion (Fig. 3D), demonstrating that protein trafficking to the apicoplast was abrogated. These data indicated that TgATG18 is critical for apicoplast biogenesis or inheritance.

IFAs were performed on parasites after Shld-1 removal from PfATG18-3HA-DD parasites using an antibody against acyl carrier protein (ACP) (Fig. 3F), which are apicoplast resident proteins. As reported earlier (24), in the wild-type situation, ACP staining revealed apicoplast branching at the onset of schizogony. Subsequently, the plastid segregated and segmented with each merozoite (Fig. 3F). In contrast, the apicoplast did not segregate to individual parasites in a significant number of Shld-1-deprived parasites (Fig. 3F), and branching was stunted. These defects were more apparent in the second and third cycles after Shld-1 removal (Fig. 3G). Similar results were obtained when IFAs were performed for another apicoplast resident protease, ClpP (data not shown). Previous studies have indicated that *Plasmodium* can survive without an apicoplast if culture medium is supplemented with isopentenyl pyrophosphate (IPP) (4). Indeed, significant restoration of parasite growth, which occurred due to PfATG18 depletion was observed (Fig. S5), which further indicated that delayed death of parasite may be due to the loss of apicoplast. Since one of the hallmarks for impaired apicoplast inheritance is loss of its genome (4, 25), quantitative PCR was performed for nuclear and apicoplast genes after ATG18 depletion in both parasites. A significant defect in apicoplast genome replication was observed in both parasites upon depletion of TgATG18 (Fig. 3E) as well as PfATG18 (Fig. 3H). Collectively, these data suggested that a role of ATG18 in apicoplast inheritance may be conserved across most *Apicomplexa*. It was interesting to note that an ATG18 homologue may not be present in *Cryptosporidium* (14).

### Interaction with 3′-PIPs is critical for Pf/TgATG18 cellular localization.

**FIG 4:**
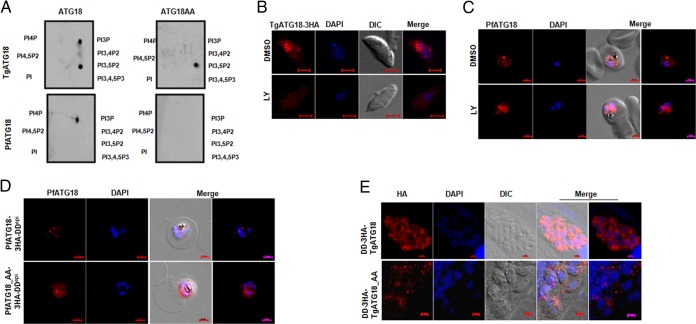
Pf/TgATG18 interact with 3′PIPs, which regulate their localization in the parasite. (A) Pf/TgATG18 interacts with PI3P via its FRRG motif. Recombinant Pf/TgATG18 or its mutant in which the FRRG motif was mutated to FAAG (ATG18-AA) was expressed as a GST fusion protein (Fig. S4A) and incubated with nitrocellulose membrane on which the indicated PIPs were spotted. Subsequently, anti-GST antibodies were used to detect the bound protein. The interaction with PI3P and PI(3,5)P2 was also confirmed by liposome binding assay (Fig. S4B and C). (B and C) LY294002 prevents targeting of ATG18 to vesicular compartments. TgATG18-3HA extracellular tachyzoites (B) or PfATG18-3HA-DD trophozoites (C) were treated with DMSO (control) or LY294002 (LY) for 40 min (B) and 8 h (C). Subsequently, the parasites were fixed, and IFA was performed using anti-HA antibody. (D) Parasite lines overexpressing PfATG18/AA-3HA-DD^epi^ were used for IFA after 120 h of Shld-1 treatment (Fig. S4E). While PfATG18 exhibited punctate localization, PfATG18-AA was diffused in the parasite cytoplasm. (E) IFA was performed after the addition of Shld-1 to DD-3HA-TgATG18 (WT or AA) parasites for 4 h. While bright punctate structures were observed in the case of TgATG18, the fluorescence was significantly weaker and fewer puncta were visible in the case of TgATG18-AA. For the purpose of illustration, an image after brightness enhancement was provided for TgATG18-AA.

The next set of experiments was performed to evaluate whether Pf/TgATG18 interacted with 3′-PIPs and dissected the role of PIPs in their cellular and parasitic function. Recombinant PfATG18 as well as TgATG18 (Fig. S4A) exhibited interaction with phosphatidylinositol 3-phosphate (PI3P) in a dot blot (Fig. 4A) as well as liposome binding assay (Fig. S4B and C). In contrast, only TgATG18, but not PfATG18, interacted with phosphatidylinositol 3,5-bisphosphate [PI(3,5)P2]. Upon mutation of the FRRG motif (Fig. S1), which has previously been demonstrated to interact with PI(3,5)P2 and PI3P (26), the PI3P binding of both Pf/TgATG18 was lost (Fig. 4A and Fig. S4B and C). Surprisingly, the TgATG18-AA mutant (TgATG18 in which the FRRG motif was mutated to FAAG [ATG18-AA]) continued to interact with PI(3,5)P2, suggesting that the FRRG motif is used for interaction with PI3P, whereas an additional independent site may be involved in PI(3,5)P2 binding (Fig. 4A and Fig. S4C). Interestingly, the lack of PI(3,5)P2 binding to PfATG18 corroborates well with the absence of both this lipid and the PI3P-5 kinase in *Plasmodium* (8, 9).

10.1128/mBio.01468-17.4 FIG S4  Interaction of recombinant GST-PfATG18 and GST-TgATG18 with PIPs and analysis of transgenic parasites ectopically expressing Pf/TgATG18 or its AA mutant. Download FIG S4, PDF file, 0.2 MB. Copyright © 2017 Bansal et al. 2017 Bansal et al. This content is distributed under the terms of the Creative Commons Attribution 4.0 International license.

A PI3-kinase inhibitor, LY294002, which depletes 3′-PIPs in both *P. falciparum* and *T. gondii* (8, 9), resulted in a loss of punctate staining of both Pf/TgATG18 (Fig. 4B and C), suggesting that PI3-kinase activity is critical for the localization of ATG18 in these parasites. Transgenic lines overexpressing the “AA” mutant of PfATG18 and TgATG18, which were fused to the DD to regulate their expression with Shld-1, were generated to further investigate the role of 3′-PIP interaction in Pf/TgATG18 cellular localization (Fig. 4D and E and Fig. S4E and S4H). To make a distinction from parasite line PfATG18-3HA-DD described above in which endogenous PfATG18 was tagged with 3HA-DD, these parasites are referred as PfATG18-3HA-DD^epi^ (epi for episomal). Wild-type PfATG18 exhibited punctate staining in parasite cytoplasm (Fig. 4D) and sometimes coincident with the food vacuole (Fig. S6) as observed for endogenous PfATG18 (Fig. 2A). The mutation of the FRRG motif resulted in diffused staining in parasite cytoplasm (Fig. 4D), suggesting that PfATG18 subcellular localization is dependent on its interaction with PI3P. Similar observations were made using a parasite line overexpressing green fluorescent protein (GFP) fused to PfATG18 (GFP-PfATG18) and the AA mutant (Fig. S4F and G). DD-HA-TgATG18 parasites exhibited punctate localization throughout the parasite cytosol; however, the number of puncta was significantly reduced in the AA mutant (Fig. 4E). It is likely that the interaction with PI(3,5)P2 allows the AA mutant (Fig. 4A) to reside in these vesicular structures, which may possess this phosphoinositide.

10.1128/mBio.01468-17.5 FIG S5  Effect of IPP on the growth of PfATG18-depleted parasites. Download FIG S5, PDF file, 0.01 MB. Copyright © 2017 Bansal et al. 2017 Bansal et al. This content is distributed under the terms of the Creative Commons Attribution 4.0 International license.

10.1128/mBio.01468-17.6 FIG S6  PfATG18 localization in *P. falciparum*. Download FIG S6, PDF file, 0.02 MB. Copyright © 2017 Bansal et al. 2017 Bansal et al. This content is distributed under the terms of the Creative Commons Attribution 4.0 International license.

**FIG 5:**
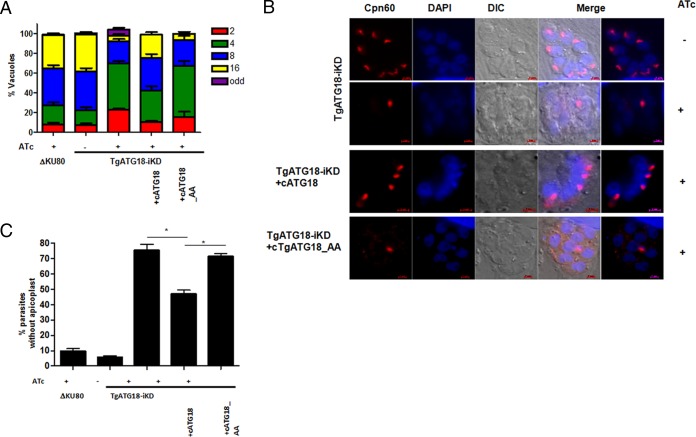
PI3P-TgATG18 interaction is critical for apicoplast biogenesis and parasite growth. (A) TgATG18-iKD parasites were complemented with either wild-type DD-3HA-TgATG18 (cATG18) or DD-3HA-TgATG18-AA (cATG18-AA). Αll parasite lines were preincubated for 48 h with ATc, and in the case of complemented lines, Shld-1 was also added, and parasites were allowed to invade fresh HFFs in the presence or absence of ATc. The number of parasites per vacuole was determined after 24 h. Values are means plus standard error of the means (error bars) from three experiments, and at least 200 vacuoles were counted for each condition. (B) IFA was performed on the indicated parasite lines pretreated with ATc for 48 h, followed by additional treatment for 2 days to detect apicoplast using an antibody against apicoplast resident protein Cpn60. In the case of complemented lines, Shld-1 was present for the duration of the experiment. (C) Parasites that have lost an apicoplast were counted from the experiment shown in panel B. Values are means plus standard errors of the means from two experiments. Values that are significantly different (*P* < 0.001) by ANOVA are indicated by a bar and asterisk.

Next, the functional relevance of PI3P-TgATG18 interaction was tested. To this end, TgATG18 or its AA mutant was overexpressed (cTgATG18/AA) in TgATG18-iKD parasites. The defect in parasite replication due to TgATG18 depletion was significantly restored upon overexpression of wild-type TgATG18 (Fig. 5A, fourth bar versus third bar [with the leftmost bar being the first bar]). In contrast, the AA mutant was unable to complement TgATG18 function (Fig. 5A, fifth bar versus third and fourth bars). The status of apicoplast inheritance was adjudged in the TgATG18 (or AA) complemented line. Cpn60 IFA revealed that the number of parasites that did not possess an apicoplast was significantly reduced in wild-type TgATG18, but not the AA mutant, complemented parasites (Fig. 5B and C). Given that TgATG18-AA does not interact with PI3P, it is reasonable to state that PI3P interaction is critical for apicoplast inheritance and parasite replication.

### ATG18 regulates ATG8 localization and membrane conjugation.

**FIG 6:**
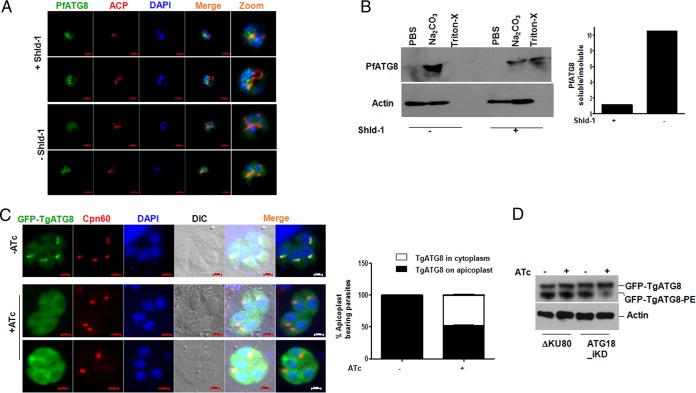
ATG18 regulates ATG8 localization and lipidation. (A) PfATG18-3HA-DD parasites were cultured for 6 days in the presence or absence of Shld-1. Subsequently, synchronized ring-stage parasites were plated, and samples of early or late schizonts were used for IFA using an antibody against ACP and an antibody against PfATG8. Shld-1 removal resulted in diffused PfATG8 localization. (B) Shld-1 was withdrawn from PfATG18-3HA-DD parasites as described above for panel A. The parasite pellet was first lysed in PBS, and the pellet was extracted with sodium carbonate (pH 11.0), followed by solubilization of the pellet in a buffer containing Triton X-100. All soluble fractions were used for Western blotting with anti-PfATG8 antibody. (Right) Densitometry of PfATG8 bands in the Western blot in the left panel was performed to determine the ratio of soluble and insoluble fractions (Triton-X soluble) in sodium carbonate. The results of one experiment representative of three independent experiments are shown. (C) TgATG18-iKD parasites expressing GFP-TgATG8 were treated with ATc for 24 h. IFA was performed to detect apicoplast protein Cpn60, and GFP fluorescence was used to localize TgATG8. TgATG8 colocalized with the apicoplast and was also observed in the cytoplasm of control parasites. Upon ATc treatment, TgATG8 was predominantly observed in the cytoplasm of parasites and was largely absent from the apicoplast. (Right) The number of apicoplast-bearing parasites that exhibited the presence (black) or absence (white) of TgATG8 on apicoplasts. (D) ΔKu80 and TgATG18-iKD parasites expressing GFP-TgATG8 were treated with ATc for 48 h. Subsequently, parasite lysates were electrophoresed on urea-SDS-polyacrylamide gel followed by Western blotting using anti-GFP antibody to detect the unmodified or PE-conjugated form of TgATG8.

In the light of present findings, it is reasonable to suggest that PI3Ks may regulate apicoplast biogenesis via Pf/TgATG18. In yeast and other organisms, ATG18 is involved in the upstream initiation step of isolation membrane formation or preautophagosomal structure (PAS) formation (11) and along with another PROPPIN family member, ATG21, is involved in ATG8 recruitment and/or conjugation to phosphatidylethanolamine (PE) via a C-terminal glycine (27, 28). Interestingly, depletion of TgATG8, which is targeted to the outer membrane of the apicoplast, also prevents apicoplast inheritance in *T. gondii* (16). While genetic disruption of PfATG8 has not been possible, it has been implicated in related apicoplast function mainly on the basis of its localization (13, 17, 29). Therefore, the role of Pf/TgATG18 in regulation of Pf/TgATG8 was evaluated. We found that PfATG8 resides in vesicular structures and while most of these vesicles were in close proximity of the branching organelle, some vesicles were coincident (Fig. 6A) as observed previously (13, 22). The depletion of PfATG18 resulted in a significant loss of punctate localization of PfATG8 along with an increase in diffused cytoplasmic localization (Fig. 6A). It has been shown previously that a significant fraction of PfATG8 is largely insoluble in sodium carbonate and can be solubilized in Triton X-100 (13), indicating its membrane association. PfATG18 depletion resulted in enhanced solubility of PfATG8 in sodium bicarbonate (Fig. 6B), suggesting that its lipidation may be impaired and that PfATG18 may regulate this process.

To evaluate the role of TgATG18 in TgATG8 regulation, GFP-TgATG8 was overexpressed in TgATG18-iKD parasites. As reported earlier (16), TgATG8 colocalized with the apicoplast and was also present in the parasite cytoplasm (Fig. 6C). Next, the effect of TgATG18 depletion on TgATG8 targeting to the apicoplast was assessed. To this end, ATc treatment was performed for 24 h to deplete TgATG18 from TgATG18-iKD parasites, which resulted in the loss of apicoplast from ~50% parasites as described above (Fig. 3B and C). While TgATG8 colocalized with Cpn60 in control parasites, it failed to localize to the apicoplast in a significant number of TgATG18-depleted parasites (Fig. 6C). In addition, a concomitant increase of TgATG8 in parasite cytoplasm was also observed. Next, we evaluated the status of TgATG8 lipid conjugation by performing urea-SDS-PAGE (15). Western blotting revealed that TgATG8 lipidation was significantly impaired upon TgATG18 depletion, as the intensity of the band corresponding to PE-conjugated TgATG8 was markedly reduced (Fig. 6D). These results suggested that Pf/TgATG18 may facilitate the membrane conjugation of Pf/TgATG8. Furthermore, the PI3K inhibitor LY294002 also prevented TgATG8 apicoplast localization (Fig. S7), which indicated that TgPI3K may be an upstream activator of this process, which fits in well with the fact that it regulates apicoplast inheritance (7).

10.1128/mBio.01468-17.7 FIG S7  Effect of LY294002 on TgATG8 localization. Download FIG S7, PDF file, 0.1 MB. Copyright © 2017 Bansal et al. 2017 Bansal et al. This content is distributed under the terms of the Creative Commons Attribution 4.0 International license.

**FIG 7:**
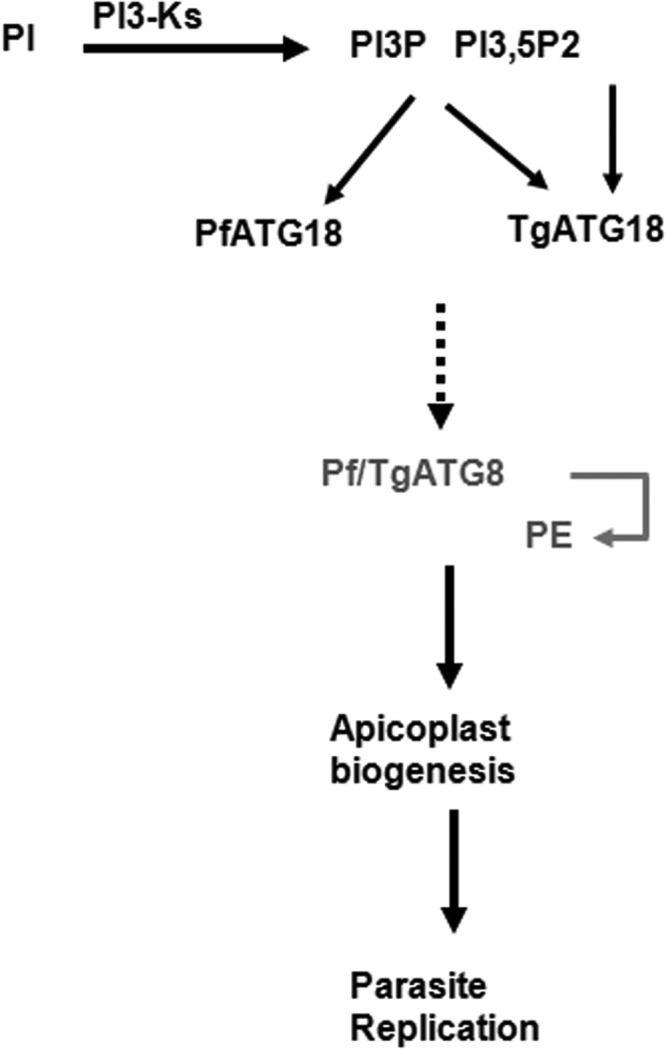
A cascade involving 3′-PIPs and ATG18 in apicoplast biogenesis in apicomplexan parasites. 3′-PIPs generated by PI3-kinase regulate apicoplast biogenesis (7), which may be mediated via Pf/TgATG18 as they interact with 3′-PIPs (Fig. 4A). These phosphoinositides target ATG18 to vesicular compartments in the parasites (Fig. 4D and E), which is critical for their function (Fig. 5A). Pf/TgATG18 may be involved in the nucleation of membrane structures to which Pf/TgATG8 conjugates (Fig. 6), which may be critical for its role in apicoplast inheritance as demonstrated for TgATG8 in *Toxoplasma* (16).

Present studies shed light on a novel cascade involved in apicoplast biogenesis (Fig. 7): 3′-PIPs generated by PI3Ks interact with Pf/TgATG18 and guide their localization to vesicular compartments and Pf/TgATG18 are critical for apicoplast biogenesis. Pf/TgATG18 depletion affected Pf/TgATG8 membrane conjugation and impaired apicoplast targeting of Pf/TgATG8. Previous studies have implicated TgATG8 in apicoplast inheritance (16). While it is possible that Pf/TgATG18 may regulate this process via Pf/TgATG8, concrete understanding of underlying mechanism is needed to firmly establish this point. One of the possibilities is that TgATG18 may facilitate a platform for recruitment of other proteins involved in ATG8 processing, facilitating the membrane conjugation of TgATG8. Subsequently, ATG8 may be targeted to the apicoplast and/or vesicles destined for the organelle.

## DISCUSSION

Phosphatidylinositol 3-kinases (PI3Ks) from *Plasmodium falciparum* and *Toxoplasma gondii* are essential for the growth of these parasites (8, 9). PI3K inhibition by LY294002 prevents *P. falciparum* proliferation under amino acid-rich conditions, as hemoglobin trafficking to the food vacuole and subsequent catabolism are impaired (10). Depletion of *T. gondii* PI3Ks (TgPI3Ks) or its inhibition by LY294002 attenuates parasite replication and apicoplast biogenesis (7, 8) of *T. gondii*. Pf/TgATG18 have emerged as key effectors of PI3Ks and PIPs in *P. falciparum* and *T. gondii* from present studies. Typically, ATG18 is involved in the initiation steps of autophagy, as it interacts with PI3P on the isolation membrane or preautophagosomal structure (PAS), which serves as a template for autophagosome formation, which is facilitated by the LC3/ATG8 conjugation system (30, 31). It is important to note that present studies were performed under nutrient-rich steady-state conditions. PfATG18 was present in vesicular compartments, which are possibly rich in PI3P. In addition, PfATG18 was found in and around the food vacuole, which fits in well with the previous demonstration of PI3P in this compartment (9, 32). Given that a food vacuole is analogous to a lysosomal compartment, it is possible that PfATG18 may have a role in autophagosome-lysosome fusion, which will be interesting to elucidate.

ATG18 was localized to endosome-like vesicular compartments in a PI3P-dependent manner even under steady-state conditions in yeast (11, 33). However, the role of ATG18 under nutrient-rich conditions is largely unclear. We did not find colocalization between ATG18 and ATG8 in both *P. falciparum* and *T. gondii*. It is likely that Pf/TgATG18 vesicles may also be endosome-like and/or trafficking vesicles, which are distinct from ATG8-rich compartments. ATG18 and ATG8 may not interact directly even in yeast, whereas another related protein, *Saccharomyces cerevisiae* ATG21 (ScATG21), interacts with ScATG8 via the D146 residue, which is absent from ScATG18 (27). Since a corresponding residue is also absent from PfATG18 and TgATG18, it may help explain the lack of interaction (data not shown) and colocalization between these proteins with Pf/TgATG8.

Pf/TgATG18 interact with 3′-PIPs via the FRRG motif, and mutation of this motif resulted in a significant loss of its vesicular localization. The FRRG mutation in TgATG18 did not result in a complete loss of punctate staining, but the number of vesicles positive for TgATG18 was significantly reduced. It is possible that the ability of the AA mutant to interact with PI(3,5)P2 allowed it to be present in some of these structures. TgATG18, but not PfATG18, exhibited interaction with PI(3,5)P2 and was independent of the FRRG motif. Interestingly, mutation of FRRG motif in ScATG18 and Hsv2 abolishes binding to both PI3P and PI(3,5)P2 (26). Therefore, TgATG18 may have a distinct mode of interaction with PI(3,5)P2. There are some differences between the PfATG18 and TgATG18 sequences (see Fig. S1 in the supplemental material), and an additional motif may exist in TgATG18 for interaction with PI(3,5)P2. It is interesting to note that PI(3,5)P2 is present in *T. gondii* (8), but not in *P. falciparum* (9), which fits in well with the fact that only *T. gondii* codes for a PI3P-5 kinase (7). The fact that the TgATG18-AA mutant, which does not interact with PI3P, did not reverse the replication and apicoplast defects highlights the importance of PI3P-TgATG18 interaction in these processes.

The activation of Vps34/PI3-kinase and subsequent generation of PI3P is critical for preautophagosome formation via ATG18, which in turn sets the stage for LC3/ATG8-mediated elongation and closure of the autophagosome (30). The conditional ablation of both TgATG18 and PfATG18 yielded surprising results, as the formation of apicoplast was impaired. The delayed death of both *P. falciparum* and *T. gondii* corroborated well with the defects in apicoplast inheritance. Previous studies indicated that TgATG8 is conjugated to the apicoplast outer membrane and that its depletion prevented apicoplast inheritance in *T. gondii* (16). Consistent with this, TgATG3 and TgATG4, which regulate TgATG8 membrane conjugation, also play a role in apicoplast homeostasis (15, 16). While genetic disruption of PfATG8 has not been reported yet, it has been speculated in apicoplast-related function on the basis of localization studies. While in some studies reasonable colocalization of PfATG8 with the apicoplast was observed, in other cases only some of the PfATG8 puncta overlapped with the apicoplast (13, 22, 29). We found PfATG8 mainly in vesicles around the apicoplast (Fig. 6A) with some puncta overlapping with the apicoplast as also observed previously. PfATG18 knockdown resulted in a significant loss of vesicular staining of PfATG8 (Fig. 6A). The regulation of ATG8 by ATG18 was clearer in *Toxoplasma*, as TgATG8 was distinctly localized on the apicoplast (16). Strikingly, TgATG18 depletion prevented TgATG8 lipidation and apicoplast targeting (Fig. 6C and D). Based on present findings, it is reasonable to conclude that PI3P-Pf/TgATG18 is important for Pf/TgATG8 lipidation and may traffic it to the apicoplast. Since we did not observe interaction between Pf/TgATG18 and Pf/TgATG8, it is difficult to pinpoint the exact mechanism by which ATG18 may regulate lipidation and apicoplast localization of TgATG8. It is likely that there are intermediate steps in this process that involve other molecules, which will be interesting to dissect.

Although further direct evidence is needed to state whether ATG18 regulates apicoplast biogenesis via ATG8, the following possibilities may exist: it is possible that Pf/TgATG18 interaction with PI3P is important for initial steps of nucleation and formation of isolation membrane-like structures as observed in yeast and mammals (11). Subsequent elongation of the membrane may be facilitated by Pf/TgATG8 lipidation, which may be relevant for apicoplast branching. Alternatively, it may regulate Pf/TgATG8 trafficking to the apicoplast where it may conjugate to its outer membrane by regulating the trafficking machinery. As proposed earlier (16), TgATG8 may regulate apicoplast inheritance by creating a link with the centrosome. However, the mechanism by which TgATG8 coordinates the apicoplast-centrosome interaction during division needs to be deciphered.

Interestingly, a recent study reported *PfATG18* single nucleotide polymorphism (SNP) in artemisinin-resistant field isolates from the China-Myanmar border, which corresponds to a T38N mutation (18). This mutation exhibited high correlation with decreased sensitivity to artemisinin (ART) and dihydroartemisinin (DHA). An independent study reported that PfATG18 was pulled down with an ART probe (34), suggesting that it may interact with this drug. Furthermore, ART-resistant Kelch13 mutations were associated with increased PfPI3K activity and PI3P production (35). Given the finding that PfATG18 is a PI3P target in malaria parasite, it is possible that there is a relationship between PfPI3K/PI3P, PfATG18, and ART resistance, which will be worth dissecting. The function of Pf/TgATG18 in apicoplast biogenesis was determined under steady-state conditions. It will also be interesting to delineate its role in nutrient-limiting conditions, which may trigger autophagy-like processes in the parasite. Preliminary data indicated that starvation may result in enhanced localization of PfATG18 to the lysosome-like compartment digestive/food vacuole (data not shown), which possibly hints at a food vacuole-related role of PfATG18 that needs further investigation.

While several core components of the autophagy pathway may be conserved in *Apicomplexa*, the autophagy machinery is rudimentary in these organisms (14). In addition, the autophagy degradation pathway has also not been demonstrated clearly in these organisms, especially in *Plasmodium*. Therefore, whether *Plasmodium* ATG genes are bona fide homologues of their yeast and mammalian counterparts is not clear, and most related functions have been unrelated to autophagy. Recent studies have implicated ATG genes in some noncanonical functions in other organisms, which includes the regulation of protein secretion and trafficking (36), but a direct role of ATG18 in organelle biogenesis seems to be unprecedented.

## MATERIALS AND METHODS

### Materials.

All the molecular biology reagents were purchased from Sigma-Aldrich. Oligonucleotides were procured from Sigma-Aldrich, and restriction enzymes were obtained from New England Biolabs, USA. Commercially available antibodies were obtained from Santa Cruz Biotechnology (mouse anti-β-actin [C4, sc-47778, conjugated to horseradish peroxidase {HRP}] [diluted 1:1,000 for Western blotting {WB}]), mouse and rabbit antihemagglutinin (anti-HA) (Y-11, sc-805) (diluted 1:500 for WB and diluted 1:200 for immunofluorescence assay [IFA]) and Roche (anti-green fluorescent protein [anti-GFP] [13.1, 11814460001] [diluted 1:200 for IFA]). *Plasmodium falciparum* antibodies were anti-*P*. *falciparum* Crt (anti-PfCrt) (MR4, rabbit, 1:400), anti-ATG8 for IFA (mouse, 1:200) (22), anti-ClpP (rabbit, 1:300), anti-glutathione *S*-transferase (anti-GST) (mouse, 1:5,000), anti-ACP (diluted 1:1,000 for IFA) (antibody was a kind gift from Geoff McFadden), and anti-*P*. *falciparum* ATG8 (anti-PfATG8) for Western blots (rabbit, 1:500) (a gift from Puran Sijwali). RPMI 1640, Dulbecco modified Eagle medium (DMEM), fetal bovine serum (FBS), and Albumax II were obtained from Invitrogen (Thermo Fisher) USA. *Toxoplasma gondii* antibodies were anti-GAP45 (rabbit, 1:10,000), anti-SAG1 (mouse, 1:5,000), anti-Cpn60 (rabbit; diluted 1:5,000, for WB and 1:200 for IFA), anti-myc (mouse cell signaling mAb9E10, 1:50), anti-MIC4 (rabbit, 1:2,000), anti-MIC2 (mouse, 1:10), anti-ROP2-4 (mouse, 1:2,500), and anti-ARO2 (rabbit 1:2,000). For immunoblot analyses, horseradish peroxidase-labeled secondary goat anti-rabbit/mouse antibodies (Molecular Probes) were used. For immunofluorescence assays, the secondary antibodies Alexa Fluor 488- and Alexa Fluor 594-conjugated goat anti-mouse/rabbit antibodies (Molecular Probes) were used. Anhydrotetracycline hydrochloride (catalog no. 37919), LY294002 (catalog no. L9908), isopentenyl pyrophosphate (IPP) (catalog no. I0503), paraformaldehyde (catalog no. 158127), Giemsa stain (catalog no. G5637), crystal violet (catalog no. C0775), mycophenolic acid (catalog no. M5255), xanthine (catalog no. X0626), and pyrimethamine (catalog no. 46706) were purchased from Sigma-Aldrich. AquaShield-1 (AS1) (0001 mM) was bought from Cheminpharma. pGEMT vector (catalog no. A137A) was purchased from Promega. 2× DyNAmo colorFlash SYBR green quantitative PCR (qPCR) master mix (catalog no. FNZ41L), SYBR green I (catalog no. S7563), DMEM (catalog no. 12800017), fetal bovine serum (catalog no. 10270106), trypsin (catalog no. 25200056), antibiotic-antimycotic (catalog no. 15240062), RPMI 1640 (catalog no. 23400021), and AlbuMAX II (catalog no.11021029) were purchased from Thermo Fisher.

Most *Toxoplasma*-related reagents, which include various vectors and antibodies, were kind gifts from Dominique Soldati-Favre, University of Geneva.

### *T*. *gondii* culture.

*T. gondii* RH strains ΔHXGPRT (37), ΔKu80 (38), or other lines generated in these backgrounds were cultured in human foreskin fibroblasts (HFFs) using DMEM supplemented with 10% fetal bovine serum and 2 mM glutamine. The induction of expression of various proteins was performed using the indicated amount of Shield-1 (Shld-1) for destabilization domain (DD) fusion proteins and 0.5 to 1.0 μg/ml of anhydrotetracycline (ATc) for TgATG18-iKD (iKD stands for inducible knockdown) parasites.

### *T. gondii* plaque and intracellular growth assays.

For plaque assays, HFF monolayer was infected with freshly egressed parasites and treated with or without ATc or Shld-1 for 7 to 10 days. The cells were fixed with paraformaldehyde (PFA) and stained with Giemsa or crystal violet.

The intracellular growth assay of TgATG18-iKD parasites was performed by pretreatment with or without ATc for 48 h unless indicated otherwise, and the parasites were cultured further for specified time with or without ATc. IFA was performed after PFA fixation using anti-GAP45 and anti-SAG1 antibody. The number of parasites per vacuole was determined, and around 200 vacuoles were counted for each condition. For DD-HA-TgATG18 and DD-HA-TgATG18-AA parasites, a similar procedure was followed except that parasites were treated with 0.5 μM Shld-1 for the indicated time.

### *T. gondii* transfection and generation of transgenic parasites.

Various plasmid DNA constructs were transfected after linearization with relevant restriction enzymes. Parasites were selected with mycophenolic acid and xanthine for hypoxanthine-xanthine-guanine phosphoribosyltransferase (HXGPRT) selection (37) and with chloramphenicol for chloramphenicol acetyltransferase (CAT) (39). Details of various transgenic parasites are provided below.

### *P. falciparum* transfection and generation of transgenic parasites.

PfATG18-3HA-DD parasites were generated in the 3D7 background by transfecting Pf*ATG18*-3HA-DD construct as described previously (40). Parasites were maintained in 0.5 μM Shld-1 from the time of transfection. Subsequently, parasites were cloned by limiting dilutions in 96-well plates. Genomic PCR was performed on individual clones, and two independent clones were used in which the integration was observed at the expected locus and the unmodified locus was not detected. The PCR product was cloned in pGEMT vector and sequenced to confirm the integration at the expected locus. More details of this parasite line and other episomal expression lines are provided below.

### *P. falciparum* growth rate assays.

*P. falciparum* lines were cultured in complete RPMI 1640 medium with 0.5% Albumax and gassed with 5% CO_2_, 3% O_2_, and 92% N_2_ at 37°C. Synchronization of parasites was achieved by treatment with 5% sorbitol. *P. falciparum* PfATG18-3HA-DD parasites were cultured in the presence of 2.5 nM WR99210 and 0.25 μM Shld-1. For knocking down *P. falciparum* ATG18 (PfATG18), Shld-1 was removed from parasites at the ring stage, and parasites were harvested for various purposes at the indicated stages. Shld-1 was either removed from cultures for 96 h prior (see Fig. S3C in the supplemental material) to performing the growth rate assay or it was removed at the beginning of the assay (day 0) (Fig. 2E). After synchronization, ring-stage parasite cultures were diluted to ~0.5 to 1% parasitemia and 2% hematocrit. The culture was divided into two halves, and Shld-1 was removed from one half, and 0.5 μM Shld 1 was added to the other half. Parasites were removed after every 24 h for preparation of thin blood smears and fluorescence-activated cell sorting (FACS) analysis.

The intraerythrocytic proliferation of parasites was measured by counting parasites from Giemsa-stained thin blood smears or by FACS, which was performed by staining infected erythrocytes with SYBR green as previously described (41).

To observe the effect of IPP on the parasite growth after knockdown of PfATG18, the growth rate assay was performed as described above except that in one of the experimental conditions, 200 µM IPP was included with Shld-1-deprived parasites and parasite growth was measured after 144 h by staining parasites with SYBR green followed by measurement of fluorescence in a BioTek plate reader (Fig. S5).

### Quantitative PCR.

Genomic DNA was isolated from parasites using a Qiagen genomic DNA isolation kit. Quantitative real-time PCR was performed in a Mastercycler RealPlex machine (Eppendorf) using primer sets designed for genes from apicoplast and nuclear genome for *Plasmodium* (SufA [apicoplast, PF3D7_0522700] and ClpP [nuclear, PF3D7_0307400]) and *Toxoplasma* (Tu [apicoplast, TGGT1_302050] and uracil phosphoribosyltransferase [UPRT] [nuclear, TGGT1_312480 {25}]). 18S rRNA was used for normalization. The amplification reaction mixture contained 50-ng template total DNA, 2× DyNAmo colorFlash qPCR master mix (Thermo Scientific) and 300 nM gene-specific primers. The expression of organelle-specific genes was defined on the basis of threshold cycle (*C*_*T*_), and the relative expression levels were determined after normalization with 18S rRNA.

### Plasmid constructs and generation of transgenic parasites.

PCR primers used for generating various constructs are listed in Table S1.

10.1128/mBio.01468-17.8 TABLE S1  List of PCR primers used. Download TABLE S1, DOC file, 0.05 MB. Copyright © 2017 Bansal et al. 2017 Bansal et al. This content is distributed under the terms of the Creative Commons Attribution 4.0 International license.

### (i) TgATG18-3HA.

An ~1.4-kb fragment was amplified using 413/414 primers from the C-terminal region of *Toxoplasma gondii* genomic DNA and cloned into LIC-HXGPRT-3HA (38). ΔKu80 parasites were electroporated with 50 μg of this plasmid digested with RsrII enzyme prior to transfection.

### (ii) TgATG18-iKD.

An ~1.3-kb fragment corresponding to the N-terminal coding sequence of *TgATG18* gene was amplified using 313/314 primers by PCR from genomic DNA and subcloned into BglII and SpeI sites of TATi-HXGPRT-tetO7Sag1MycTag vector (20). The 5′ flanking untranslated region (UTR) upstream of the *TgATG18* promoter (1,310 bp) was PCR amplified by 315/316 primers and cloned into the NcoI and BamHI sites in TATi-HXGPRT-tetO7Sag1-MycNtATG18 vector. The resulting plasmid pATG18-TATi-HXGPRT-tetO7Sag1-MycNt*TgATG18* was transfected in ΔKu80 parasites by electroporation of 50 μg of this construct (linearized with both NcoI and NotI prior to transfection). Parasites were subsequently subjected to mycophenolic acid (MPA) and xanthine selection. The drug-resistant parasites were cloned by limiting dilutions, and clones were screened by PCR to detect the presence of endogenous or recombined locus.

### (iii) DD-HA-TgATG18 (WT or AA) parasites.

To generate the DDHA-*TgATG18*, *TgATG18* cDNA was amplified by PCR using Phusion DNA polymerase and primers 420/421 and cloned into the pGEMT vector. Site-directed mutagenesis was used to mutate the FRRG motif to FAAG and was cloned into the pCTDD-HA construct between the EcoRV and PstI sites. One hundred micrograms of the circular plasmid was transfected in ΔHXGPRT as well as TgATG18-iKD (for complementation with wild-type [WT] *T*. *gondii* ATG18 [TgATG18] or TgATG18-AA mutant) strains, and selection was done using chloramphenicol. DD/HA-TgATG18 expression was induced by the addition of 0.1 μM Shld-1.

### (iv) GFP-TgATG8.

GFP-TgATG8 cDNA was PCR amplified using the indicated primers and cloned into the pTub8-HX vector in EcoRI/PacI sites. For transient transfections, TgATG18-iKD parasites were used for electroporation with 100 μg of circular plasmid pTub8-GFP-TgATG8-HX. For stable GFP-TgATG8 expression, HXGPRT selection cassette was replaced with dihydrofolate reductase (DHFR), parasites were selected with pyrimethamine for three passages, and a single clonal population obtained by limiting dilution cloning was used for further studies.

### (v) PfATG18-3HA-DD.

A silent point mutation was introduced using site-directed mutagenesis to disrupt the KpnI restriction site in *PfATG18* to facilitate cloning in various vectors. A 3′ fragment (1,008 bp) of *PfATG18* was PCR amplified using primers 332/333 from *P. falciparum* genomic DNA and cloned into SacII and KpnI restriction sites in the pHADD vector to tag the *PfATG18* locus with 3×HA-DD. One hundred micrograms of plasmid DNA was used for the electroporation in sorbitol-synchronized ring-stage 3D7 parasites. Transgenic parasites were maintained on 0.5 µM Shld-1, and 2 nM WR99210 was cycled on/off for 4 cycles with 2 weeks of each on or off cycle. The genotyping of drug-selected parasites revealed a mixed population of parasites containing both the wild type and integrants. Subsequently, parasites were cloned to obtain pure populations of recombinant parasites. The parasites were genotyped by performing PCR, and sequencing of the PCR products was performed to confirm integration at the desired locus.

### (vi) GFP-PfATG18 (WT or AA mutant).

For episomal expression, PfATG18 was PCR amplified with primers Pf3F/Pf4R (F stands for forward, and R stands for reverse) and inserted in AvrII and XhoI restriction sites in the NpARL vector (N-terminal GFP overexpression) (42). Site-directed mutagenesis was used to mutate the FRRG motif to FAAG to obtain PfATG18-AA, and subsequently cloned into the N-pARL vector in AvrII and XhoI sites. One hundred micrograms of plasmid DNA was used for electroporation in 8% sorbitol-synchronized ring-stage 3D7 parasites. Transgenic parasites were selected using 10 nM WR99210.

### (vii) PfATG18-3HA-DD^epi^.

For episomal expression of PfATG18 or its AA mutant with a C-terminal 3HA-DD using an hsp86 promoter, PfATG18 cDNA and PfATG18-AA mutant were cloned into the pHADD vector between the XhoI and KpnI restriction sites using 407/408 primers. One hundred micrograms of plasmid DNA was used for the electroporation in sorbitol-synchronized ring-stage 3D7 parasites. Transgenic parasites were selected using 10 nM WR99210.

### Immunofluorescence assays.

For immunofluorescence assays of *T. gondii*, human foreskin fibroblasts (HFFs) seeded on coverslips were infected with freshly egressed parasites. Intracellular parasites cultured for the indicated times were fixed with 4% PFA or 4% PFA and 0.05% glutaraldehyde (PFA/GA) in phosphate-buffered saline (PBS) followed by incubation with various primary antibodies and processed as described previously (43). For IFA on *P. falciparum*, 4% paraformaldehyde and 0.0075% glutaraldehyde in PBS (pH 7.4) was used for fixation, and the parasites were permeabilized with 0.1 to 0.2% Triton X-100 and blocked with 3% bovine serum albumin (BSA) for 30 min at room temperature. The parasites were incubated with relevant antibodies at 4°C, washed with PBS, and incubated with Alexa Fluor 488 or 594 (488/594)-labeled secondary antibodies (Invitrogen) at room temperature. Following washing, mounting medium containing 4′,6′-diamidino-2-phenylindole (DAPI) was used to stain the nucleus. The stained parasites were visualized using AxioImager Z1 microscope or LSM700 confocal microscope (Carl Zeiss). Z stacking during image acquisition and processing of images was done using AxioVision 4.8.2 software. Z-stacks that best represented the immunolocalization were used for illustrations in the figures. The constrained iterative algorithm in the deconvolution module of AxioVision Software was used for some IFAs. Adobe Photoshop software was also used for preparing images for figures.

### Immunoblotting.

Freshly egressed *T. gondii* parasites were used for the preparation of lysate, which was done in a buffer containing 10 mM Tris (pH 7.5), 100 mM NaCl, 5 mM EDTA, 1% Triton X-100, and Complete protease inhibitor mixture (Roche Applied Science) protease inhibitor cocktail. In the case of *P. falciparum*, parasites were first separated from the infected red blood cells (iRBC) by saponin lysis (10). Following protein estimation, SDS-PAGE was performed, followed by transfer of proteins to nitrocellulose membranes. Immunoblotting was performed using various primary antibodies and antisera, and HRP-labeled anti-rabbit IgG WestPico or West Dura enhanced chemiluminescence (ECL) substrate (Pierce) was used to develop blots following the manufacturer’s instructions.

### Lipidation of ATG8.

The lipidation status of GFP-TgATG8 was assessed by using a previously published protocol (15). Briefly, parasite lysates were separated on 10 or 15% SDS-polyacrylamide gels with 6 M urea, and the GFP-ATG8-PE forms of GFP-ATG8 were detected by standard Western blot analysis using anti-GFP antibody.

### Solubility of PfATG8.

Parasite protein fractionation was performed as described previously (13) with some modifications. Briefly, the parasite pellet was suspended in 200 μl PBS containing protease inhibitor cocktail (Roche), and subjected to lysis using a syringe on ice followed by centrifugation at 16,000 × *g* for 1 h at 4°C. The supernatant that contained soluble cytosolic proteins was transferred to a fresh tube. The pellet was resuspended in 100 mM sodium carbonate (pH 11.5) with protease inhibitor cocktail and subjected to further lysis and centrifugation. The remaining pellet obtained was solubilized in PBS containing 2% Triton X-100. All soluble fractions were normalized and used for Western blotting with anti-PfATG8 antibody.

### **Effect of** LY294002 **on TgATG8.**

Intracellular parasites expressing GFP-TgATG8 were grown for 24 h and treated with 50 μM LY294002 or dimethyl sulfoxide (DMSO) for 2 h. Cells were fixed using 4% PFA in PBS, and IFA was performed as described earlier using Cpn60 antibody.

### Expression of recombinant Tg/PfATG18 or AA mutant.

TgATG18 or PfATG18 cDNA was PCR amplified and cloned in BamHI and NotI restriction sites of the pGEX4TI vector for recombinant protein expression and PIP binding assay. The mutation of the FRRG motif to FAAG was performed by site-directed mutagenesis. Recombinant proteins were expressed in *Escherichia coli* BL-21 RIL strain. The cultures were grown at 37°C until an optical density at 600 nm of 0.6 to 0.8 was reached and induced with 1 mM isopropyl-β-d-thiogalactopyranoside (IPTG) at 18°C for 16 h. Cells were pelleted by centrifugation at 4,000 rpm for 10 min at 4°C and suspended in 50 mM Tris-HCl (pH 7.4), 2 mM EDTA, 1% Triton X-100, 1 mM dithiothreitol (DTT), and protease inhibitors. Samples were sonicated for 10 cycles, and supernatant was used to affinity purify recombinant protein using glutathione Sepharose beads.

### Dot blot assay for phosphoinositide binding.

Various phosphoinositides were spotted onto nitrocellulose membranes at a concentration of 100 µg and air dried for 1 h. The membrane was blocked using 3% BSA in Tris-buffered saline with Tween 20 (TBST) buffer (50 mM Tris-HCl [pH 7.4], 150 mM sodium chloride, 0.1% Tween 20) for 2 h, followed by overnight binding to 0.5 µg/ml of recombinant protein GST-PfATG18/TgATG18 or glutathione *S*-transferase (GST) at 4°C. The membrane was washed three times in TBST buffer and probed with anti-GST antibody for 2 h at 25°C. Subsequently, the membrane was washed with TBST buffer three times and incubated with horseradish peroxidase (HRP)-conjugated anti-mouse IgG, and signal was detected using chemiluminescence.

### Liposome binding assay.

Phosphatidylcholine (1.5 mg), phosphatidylethanolamine (PE) (1.4 mg), and phosphoinositides (110 µg) were mixed at 4°C and dried under N_2_ stream. The dried lipid mixture was resuspended in 100 µl of 10 mM HEPES (pH 7.2), 100 mM NaCl, 2 mM EGTA, and 0.1 µg/ml BSA by bath sonication for 20 min. Two micrograms of GST fusion protein was incubated with liposome vesicles at 30°C for 20 min, followed by ultracentrifugation at 85,000 rpm for 20 min at 4°C. The pellet and supernatant fractions were electrophoresed on 10% SDS-polyacrylamide gels, and immunoblotted using anti-GST antibody.
