# Supplementary material for: Autophagy-Related Protein ATG18 Regulates Apicoplast Biogenesis in Apicomplexan Parasites
Source: mBio. 2017 Oct 31;8(5):e01468-17. doi: 10.1128/mBio.01468-17 (PMC5666157; doi:10.1128/mBio.01468-17)
Supplement: FIG S1 [file mbo005173561sf1.pdf]

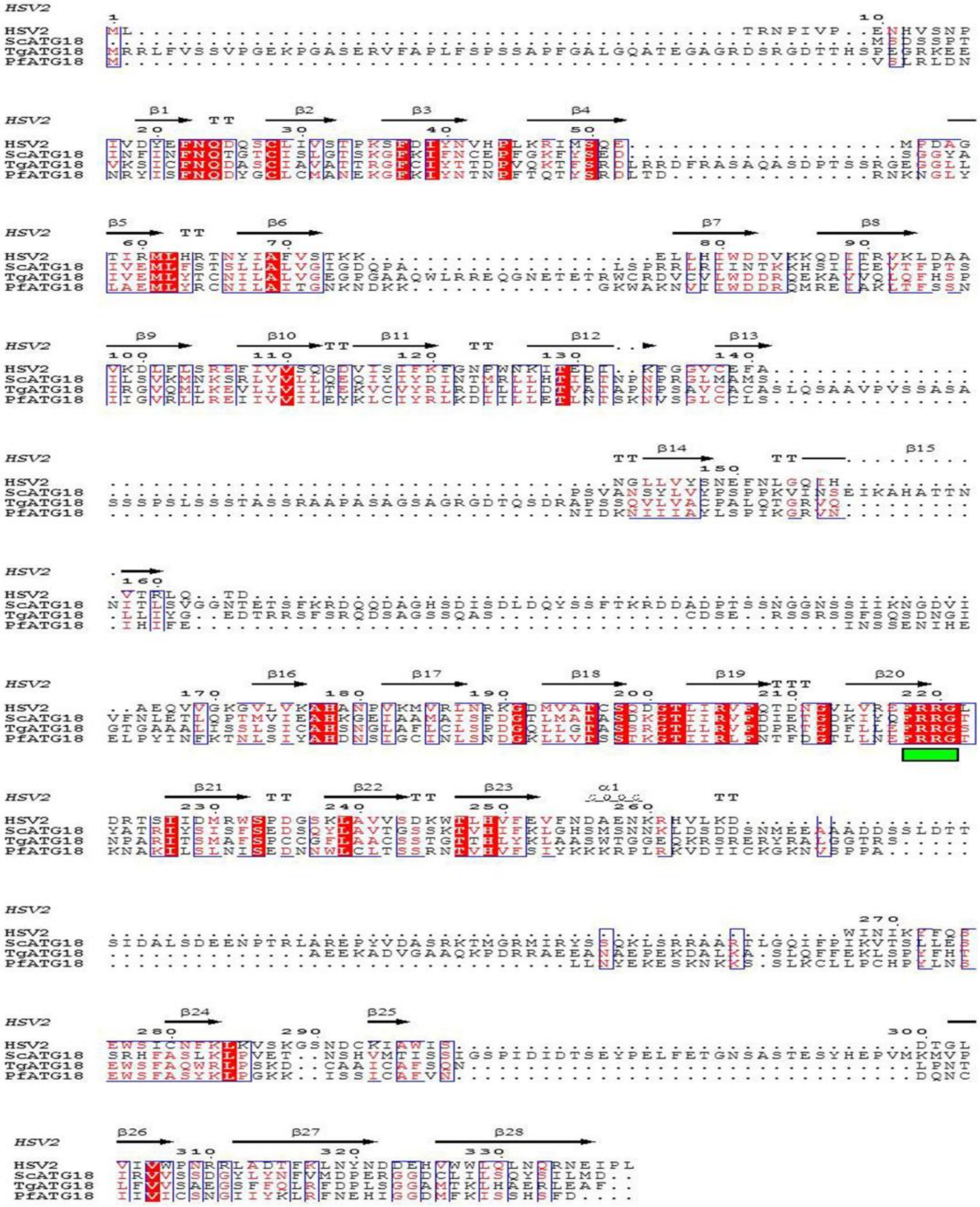

Supplementary Figure S1: Sequence comparison of PfATG18 and TgATG18 with ScATG18. Structure based sequence alignment of Pf/TgATG18 with ScATG18 and Hsv2 was performed using the crystal structure of Hsv2 [(PDB ID 4AV9; and (Krick et al., 2012)]. Multiple sequence alignment was done using PROMALS3D and visualized by ESPirit3.0. Approximate position of the secondary structure elements is indicated and the FRRG motif is indicated by a green bar.
