# Supplementary material for: Autophagy-Related Protein ATG18 Regulates Apicoplast Biogenesis in Apicomplexan Parasites
Source: mBio. 2017 Oct 31;8(5):e01468-17. doi: 10.1128/mBio.01468-17 (PMC5666157; doi:10.1128/mBio.01468-17)
Supplement: FIG S2 [file mbo005173561sf2.pdf]

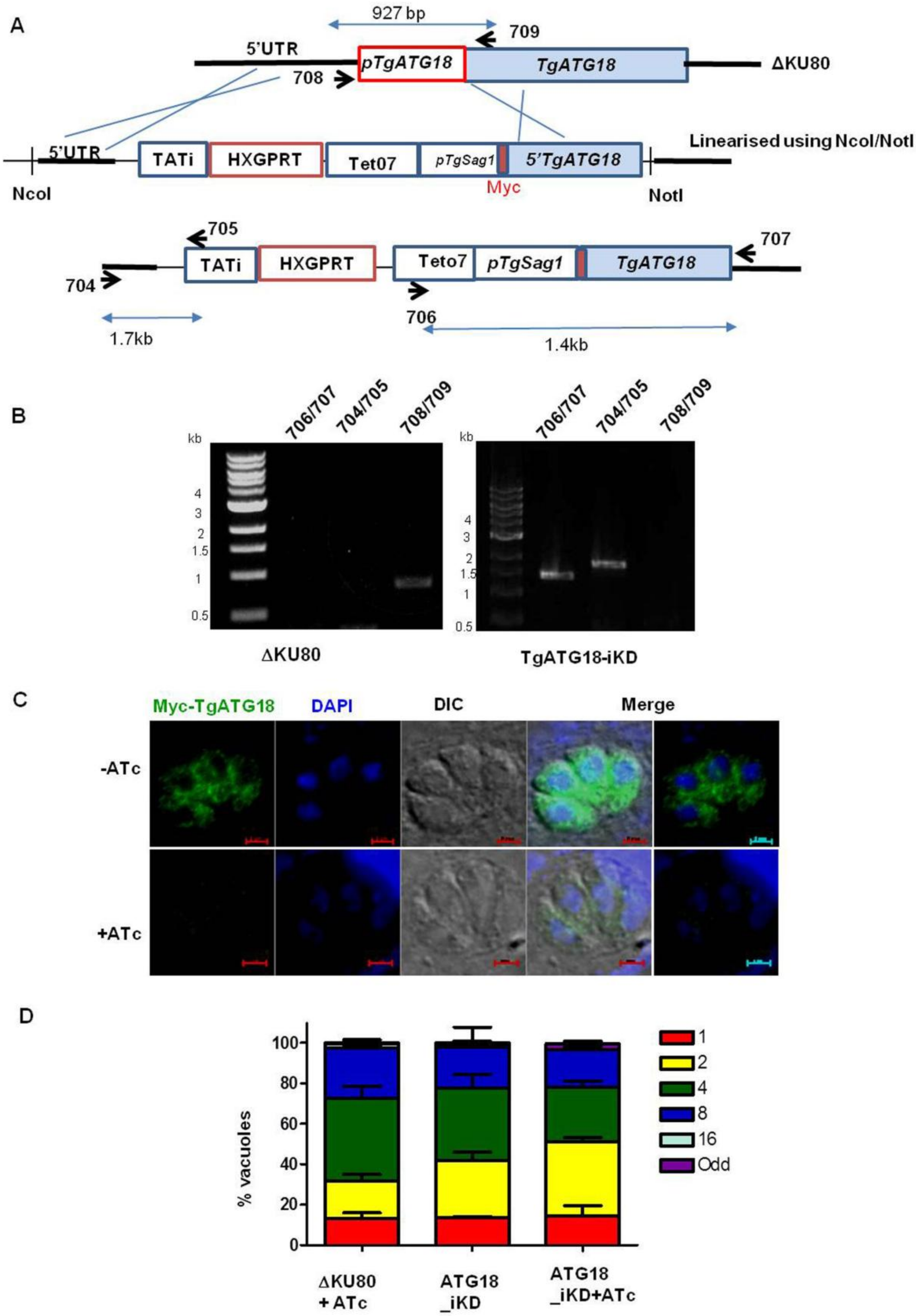
A
927 bp
709
5'UTR
pTgATG18
TgATG18
ΔKU80
708
5'UTR
TATi
HXGPRT
Tet07
pTgSag1
5'TgATG18
Linearised using NcoI/NotI
NcoI
Myc
NotI
705
707
TATi
HXGPRT
Teto7
pTgSag1
TgATG18
704
706
1.7kb
1.4kb
B
706/707
704/705
708/709
kb
4
3
2
1.5
1
0.5
ΔKU80
TgATG18-iKD
C
Myc-TgATG18
DAPI
DIC
Merge
-ATc
+ATc
D
% vacuoles
100
80
60
40
20
0
1
2
4
8
16
Odd
ΔKU80 + ATc
ATG18 _iKD
ATG18 _iKD+ATc

Supplementary Figure S2: Generation and analysis of TgATG18-iKD parasites

A. Schematic representation of the strategy used to generate TgATG18-iKD parasites. A plasmid construct that allowed insertion of transactivator TATi-1 and replacement of *TgATG18* promoter with the 7tet-Op SAG1 (TetO7) inducible promoter was introduced by homologous recombination in TgATG18 locus. In addition, a Myc tag was inserted at the N-terminus of TgATG18. After transfection, the drug selected parasites were cloned by limiting dilution.

B. Genotyping of *TgATG18*-iKD parasites. The data shown is for one of the two independent clones obtained, which was used for the reported studies. PCR amplification of the unmodified and recombined locus was performed using indicated primers; primer 708/709 for endogenous, 704/705 for 5'-integration and 706/707 for 3'-integration of the recombined locus. PCR products of expected size were obtained and the endogenous locus was absent in the transgenic TgATG18-iKD parasites. The amplicon obtained by primer set 704/705 and 706/707 was cloned and sequenced, which confirmed the integration at the expected site.

C. IFA was performed on TgATG18-iKD parasites in which Myc-tag is fused to TgATG18 (panel A) using anti-myc antibody after 48h of ATc treatment.

D. Intracellular replication of TgATG18-iKD parasites after one cycle of ATc treatment. TgATG18-iKD or ΔKu80 were left untreated or treated with ATc and the number of parasites per vacuole was determined after 24h. Data represent SEM+/- SE, n=3 and at least 200 vacuoles were counted for each condition.
