# Supplementary material for: Autophagy-Related Protein ATG18 Regulates Apicoplast Biogenesis in Apicomplexan Parasites
Source: mBio. 2017 Oct 31;8(5):e01468-17. doi: 10.1128/mBio.01468-17 (PMC5666157; doi:10.1128/mBio.01468-17)
Supplement: FIG S3 [file mbo005173561sf3.pdf]

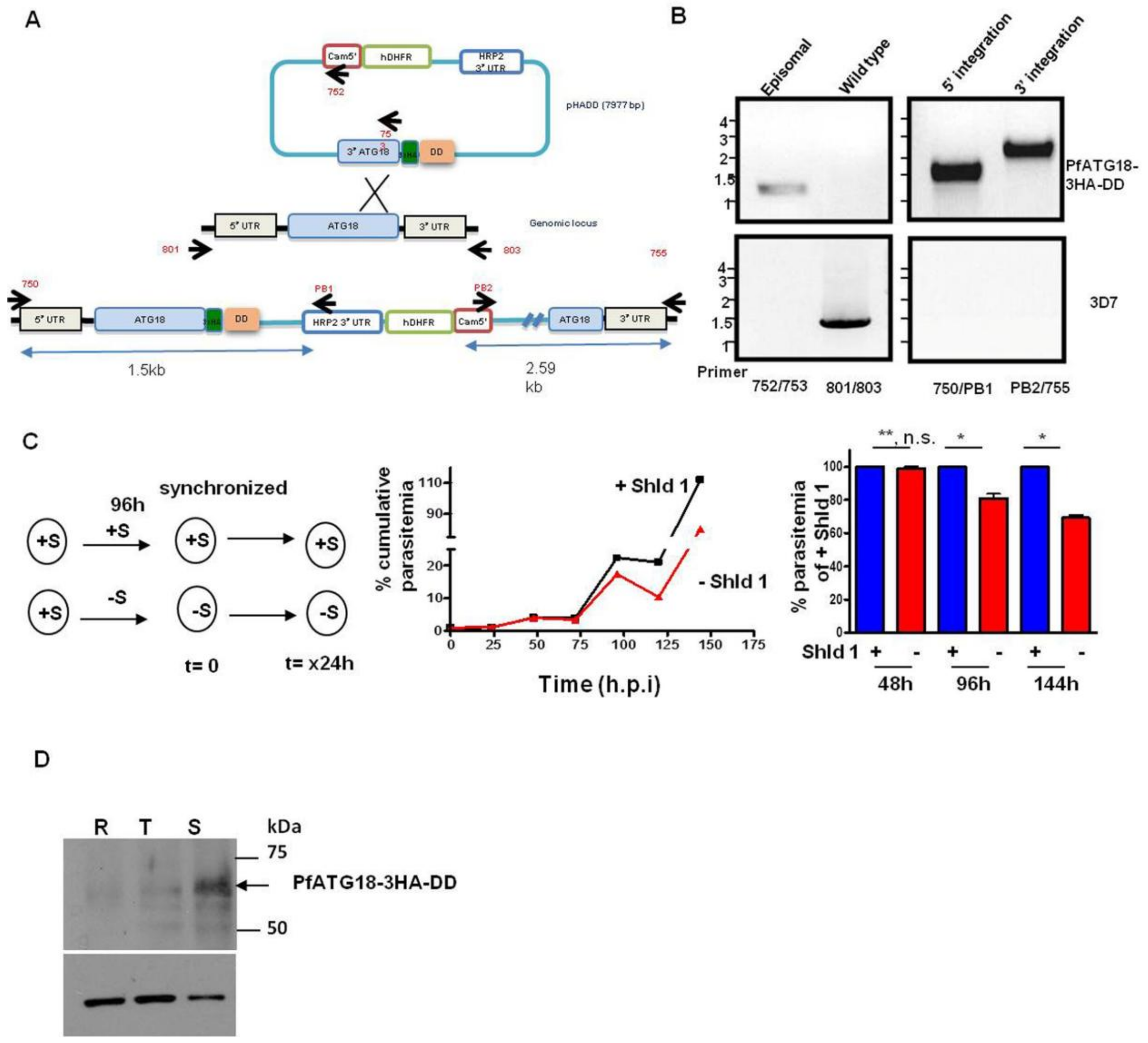

Supplementary Figure S3: Generation and analysis of PfATG18-3HA-DD parasites.

A. Schematic illustrates the targeting plasmid for the integration in the *PfATG18* locus. A fragment corresponding to 3'-end of the gene was cloned in pHADD vector upstream of the 3xHA tag and the DD domain to facilitate single crossover homologous recombination. The location of primers used for detecting the episome (752/753), unmodified locus (801/803), 5'-integration (750/PB1) and 3'-integration (PB2/755) is indicated. This plasmid construct was transfected in 3D7 strain of *P. falciparum* and parasites were selected with WR99210 as the

hDHFR coding sequence was present in the targeting plasmid. Several alternating cycles of culture with and without drug were used and parasites were always maintained in Shld-1.

B. PCR amplification was performed using genomic DNA from 3D7/WT or one of the clones of PfATG18-3HA-DD parasites. The location of primers used is indicated in panel A. PCR products of the expected size were obtained and transgenic parasites did not show the presence of the unmodified wild type *ATG18*. The PCR product for 5'-integration and 3'-integration was sequenced to confirm the site of integration.

C. Effect of PfATG18 depletion on parasite growth.

Shld-1 was withdrawn for 96h from one half of cultures of PfATG18-3HA-DD parasites. Subsequently, synchronized rings were plated (time = 0h) and cultured ± Shld-1. Growth was monitored by analyzing Giemsa stained thin blood smears or by FACS at indicated time points. *Right panel*, the SEM parasitemia at 48, 96 and 120h for 3 independent experiments similar to the one described in the left panel [SEM+/- SE , n=3 (t-test ,*, p<0.001; **, n.s.)].

D. Lysates were prepared from cultures of PfATG18-3HA-DD parasites synchronized at ring (R), trophozoite (T) and schizonts (S), which was used for western blotting with anti-HA antibodies.
