# Supplementary material for: Autophagy-Related Protein ATG18 Regulates Apicoplast Biogenesis in Apicomplexan Parasites
Source: mBio. 2017 Oct 31;8(5):e01468-17. doi: 10.1128/mBio.01468-17 (PMC5666157; doi:10.1128/mBio.01468-17)
Supplement: FIG S4 [file mbo005173561sf4.pdf]

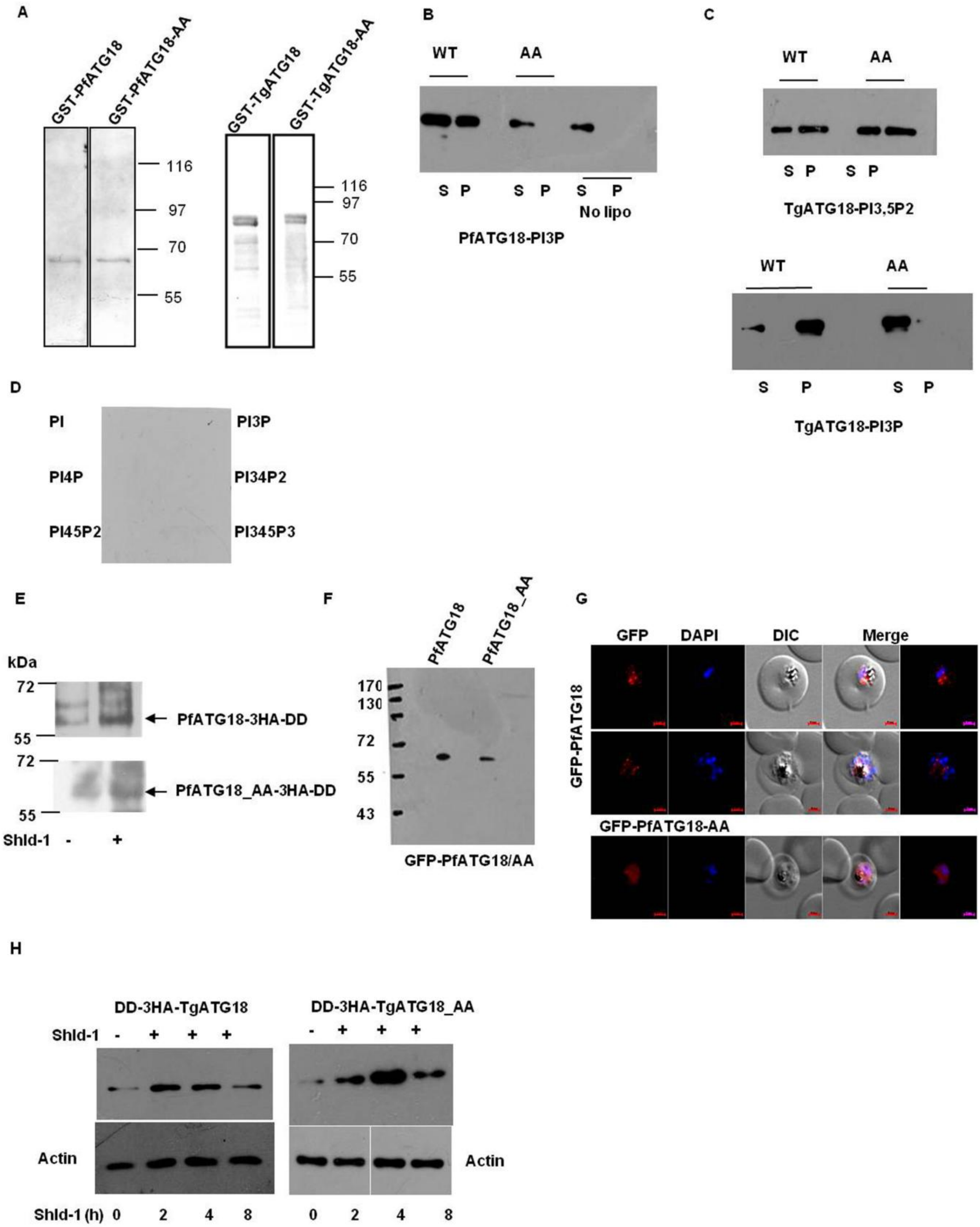

A
GST-PfATG18
GST-PfATG18-AA
116
97
70
55
GST-TgATG18
GST-TgATG18-AA
116
97
70
55
B
WT
AA
S P
S P
S P
No lipo
PfATG18-PI3P
C
WT
AA
S P
S P
TgATG18-PI3,5P2
WT
AA
S P
S P
TgATG18-PI3P
D
PI
PI3P
PI4P
PI34P2
PI45P2
PI345P3
E
kDa
72
55
PfATG18-3HA-DD
72
55
PfATG18_AA-3HA-DD
Shld-1 - +
F
PfATG18
PfATG18_AA
170
130
72
55
43
GFP-PfATG18/AA
G
GFP
DAPI
DIC
Merge
GFP-PfATG18
GFP-PfATG18-AA
H
DD-3HA-TgATG18
DD-3HA-TgATG18_AA
Shld-1 - + + +
- + + +
Actin
Actin
Shld-1 (h) 0 2 4 8
0 2 4 8

Supplementary Figure S4: Interaction of recombinant GST-PfATG18 and GST-TgATG18 with PIPs and analysis of transgenic parasites ectopically expressing Pf/TgATG18 or its AA mutant.

A. Recombinant GST-PfATG18 and GST-TgATG18 or their AA mutants were expressed and purified using affinity chromatography.

B and C. Liposomes incorporated with PI3P or PI3,5P2 were prepared. A control in which phosphatidylcholine and PIPs were excluded was also used (No lipo). Recombinant GST-PfATG18 (B) and GST-TgATG18 (C) or their "AA" mutant were incubated with PI3P or PI(3,5)P2 liposomes. The pellet comprising of the liposome (P) was separated from supernatant (S) that contained the unbound protein. Western blotting was performed with anti-GST antibody to detect the liposome-bound protein. While both Pf and TgATG18 (and not their AA mutant) exhibited interaction with PI3P, both TgATG18 and its AA mutant interacted with PI(3,5)P2. These data were consistent with the dot blot assay (Fig. 4A).

D. GST was used as a control in dot blot assays described in Fig. 4, it did not exhibit interaction with any PIP.

E and F: Overexpression of Wild Type or AA mutant of PfATG18. 3D7 parasites were transfected with a plasmid construct generated by cloning either *PfATG18* or its *PfATG18*-AA mutant in pARL vector with N-terminus GFP (F) or pHADD vector with C-terminus fused to DD and 3xHA (3HA-DD) (E). Drug resistant parasites were used for western blotting anti-GFP (F) or anti-HA antibodies (E).
