# Supplementary material for: Autophagy-Related Protein ATG18 Regulates Apicoplast Biogenesis in Apicomplexan Parasites
Source: mBio. 2017 Oct 31;8(5):e01468-17. doi: 10.1128/mBio.01468-17 (PMC5666157; doi:10.1128/mBio.01468-17)
Supplement: FIG S5 [file mbo005173561sf5.pdf]

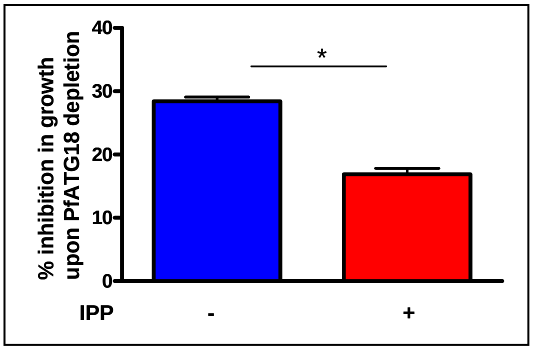

Supplementary Figure S5: Effect of IPP on growth of PfATG18 depleted parasites.

PfATG18-3HA-DD parasites grown in the presence or absence of Shld-1 as described in main text. In addition, in one set of Shld-1 (-) parasites 200 µM IPP was added. Parasite growth was measured using a SYBR-green based assay after 144h. % inhibition in growth of Shild-1 deprived parasites in comparison to Shld-1 treated parasites (100%) is represented [SEM+/- SE , n=3 (t-test ,*, $p<0.05$)].
