# Supplementary material for: Autophagy-Related Protein ATG18 Regulates Apicoplast Biogenesis in Apicomplexan Parasites
Source: mBio. 2017 Oct 31;8(5):e01468-17. doi: 10.1128/mBio.01468-17 (PMC5666157; doi:10.1128/mBio.01468-17)
Supplement: FIG S6 [file mbo005173561sf6.pdf]

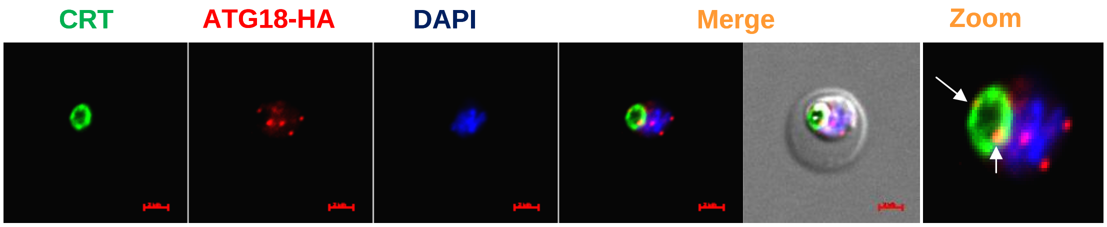

Supplementary Figure S6: PfATG18 localization in *P. falciparum*.

IFA was performed on parasites PfATG18-3HA-DD^{epi} overexpressing PfATG18 using anti-HA and anti-CRT antibody, which stains the food vacuole membrane. Some PfATG18 positive punctii were found in close proximity of FV (indicated by arrows).
