# Supplementary material for: Autophagy-Related Protein ATG18 Regulates Apicoplast Biogenesis in Apicomplexan Parasites
Source: mBio. 2017 Oct 31;8(5):e01468-17. doi: 10.1128/mBio.01468-17 (PMC5666157; doi:10.1128/mBio.01468-17)
Supplement: FIG S7 [file mbo005173561sf7.pdf]

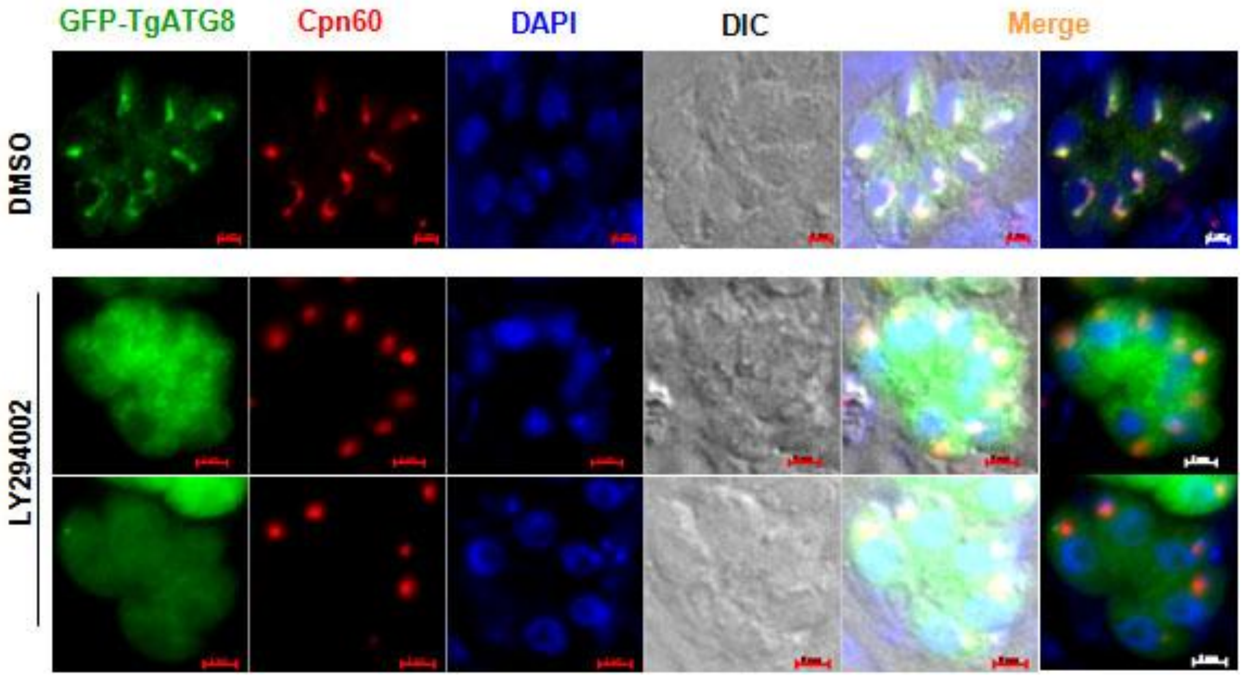


**Supplementary Figure S7:** Effect of LY294002 on TgATG8 localization

Parasites expressing GFP-TgATG8 were grown for 24 hours followed by treatment with 50 μM LY294002 for 2 hours. Cells were fixed using 4% PFA in PBS and IFA was performed as described earlier using Cpn60 antibody. LY294002-treatment prevented TgATG8 localization on apicoplast.
