# Supplementary material for: Autophagy-Related Protein ATG18 Regulates Apicoplast Biogenesis in Apicomplexan Parasites
Source: mBio. 2017 Oct 31;8(5):e01468-17. doi: 10.1128/mBio.01468-17 (PMC5666157; doi:10.1128/mBio.01468-17)
Supplement: TABLE S1 [file mbo005173561st1.doc]

**Table S1: List of PCR primers used**

***PfATG18 related***

| Construct | Forward (5’-3’) | Reverse (5’-3’) |
| --- | --- | --- |
| *PfATG18*-3HA-DD | 332  CTCCGCGGTAACTGATAAAATAAGAATGGTTT | 333  GGGTACCGTCAAAACTGTGTGATGATA |
| *PfATG18*-3HA-DD: 5’ integration | 750  TGGATGTATAAGTAAATATGT | PB1  ACGAACATTAAGCTGC |
| *PfATG18*-3HA-DD: 3’ integration | PB2  TACATTGTTTAATACTACTA | 755  TATGCGGAAATTAATATT |
| *PfATG18*-3HA-DD: Episome | 752  TAAACCAATAGATAAAATTTGTAGAGAA | 753  AACTGTGTGATGATATTTTAAACATAT |
| *PfATG18*-3HA-DD: WT locus | 801  ATGGTATCATTAAGATTAGAT | 803  TCTTATTTTCTTTTCATCCAT |
| GFP-*PfATG18*/AA | Pf3F  GGCCCTAGGATGGTATCATTAAGATTAGATAATAATAG | Pf4R  CCCTCGAGATCAAAACTGTGTGATGATAT |
| *PfATG18*-3HA-DD^epi^ | 407  GGCCTCGAGATGGTATCATTAAGATTAGATAATAATAG | 408  CGCGGTACCGCTTAATCAAAACTGTGTGATGA |
| *PfATG18* FRRG mutation to FAAG | 176  ATTATTAAATGAATTCGCAGCAGGTACTAAAAA | 177  TTTTTAGTACCTGCTGCGAATTCATTTAATAAT |
| *PfATG18*-pGeX-4T1 | 174  GGCGGATCCATGGTATCATTAAGATTAGATAATAATAG | 175  CGCGCGGCCGCTTAATCAAAACTGTGTGATGA |

***TgATG18*** related

| Oligo | Forward (5’-3’) | Reverse (5’-3’) |
| --- | --- | --- |
| LIC-HXGPRT-*TgATG18*-3HA | 413 TACTTCCAATCCAATTTAATGCAGCAATCCTGCGCGAATCACCTCGAT | 414  TCCTCCACTTCCAATTTTAGCAAACGCTTCCAGACGCTCTGCATGCAGTT |
| TgATG18-iKD: N-Terminal flank cloning | 313 CTAGATCTGGCGTCTCTTTGTCTCCTC | 314  CACTAGTCTCTGTTCGTTTCCGAGGC |
| TgATG18-iKD: 5’UTR flank  Cloning | 315 GCCATGGCCTTAAGGGAAAAGGCCGCCTC | 316  AACTGCAGCTAAAACGCTTCCAGACGCTCT |
| TgATG18-iKD: 5’ integration | 704  GTTTTCCAGTGTAAATGAAAC | 705  AGCAGAGTTGATGACTTT |
| TgATG18-iKD: 3’ integration | 706  GGATTTCCGTACCTCTC | 707  TTGGATGTCGGTTTCG |
| TgATG18-iKD: WT locus | 708 TCTCCCGTCCTCTTCCCTGC | 709  TGAAGGCCGGCGACAGAAAGA |
| *DD-HA-TgATG18* overexpression | 420  CGATATCATGAGGCGTCTCTTTGTCTCCT | 421  AACTGCAGCTAAAACGCTTCCAGACGCTCT |
| *GFP-TgATG8* | cttgaattccctttttcgacaaaATGAGTAAAGGAGA | gtgattaaTTAATTACCCCAGAGTGTTC |
| *TgATG18* FRRG mutation to FAAG | 178  ATTTCCTCTTGGAGTTCGCGGCAGGGA GCAATCCTGCGCGA | 179  TCGCGCAGGATT GCTCCCTGCCGC GAACTCCAAGAGGAAAT |
| *TgATG18*-pGeX-4T1 | 195  GGCGGATCCATGAGGCGTCTCTTTGTCTCCT | 196  CGCGCGGCCGCCTAAAACGCTTCCAGACGCTCT |

Real Time PCR Primers for apicoplast and nuclear genes:

| Oligo | Forward (5’-3’) | Reverse (5’-3’) |
| --- | --- | --- |
| *TgTu* | TCTATTGCAATGGAAAAAGGTATG | TCAATGGTAGAGCAAAGGACTG |
| *TgUPRP* | ACTGCGACGACATACTGGAGAAC | AAGAAAACAAAGCGGAACAACAA |
| *PfSuf A* | ATTGGATACAAGTTGAAGTAGG | CTACCAGTATCAGCTATTTGC |
| *PfClp P* | AACGTACATAATAGCTCATCAG | TGGTATGTTGTATCATTCCTTG |
| *18SRNA* | GCTGACTACGTCCCTGCCC | ACAATTCATCATATCTTTCAATCGGTA |
